# Adaptive and Innate Immunity in Psoriasis and Other Inflammatory Disorders

**DOI:** 10.3389/fimmu.2019.01764

**Published:** 2019-07-26

**Authors:** Michael P. Schön

**Affiliations:** Department of Dermatology, Venereology and Allergology, University Medical Center Göttingen, Göttingen, Germany

**Keywords:** psoriasis, adaptive immunity, innate immunity, autoimmune disease, skin—immunology

## Abstract

Over the past three decades, a considerable body of evidence has highlighted T cells as pivotal culprits in the pathogenesis of psoriasis. This includes the association of psoriasis with certain MHC (HLA) alleles, oligoclonal expansion of T cells in some cases, therapeutic response to T cell-directed immunomodulation, the onset of psoriasis following bone marrow transplantation, or induction of psoriasis-like inflammation by T cells in experimental animals. There is accumulating clinical and experimental evidence suggesting that both autoimmune and autoinflammatory mechanisms lie at the core of the disease. Indeed, some studies suggested antigenic functions of structural proteins, and complexes of self-DNA with cathelicidin (LL37) or melanocytic ADAMTSL5 have been proposed more recently as actual auto-antigens in some cases of psoriasis. These findings are accompanied by various immunoregulatory mechanisms, which we increasingly understand and which connect innate and adaptive immunity. Specific adaptive autoimmune responses, together with our current view of psoriasis as a systemic inflammatory disorder, raise the question of whether psoriasis may have connections to autoimmune or autoinflammatory disorders elsewhere in the body. While such associations have been suspected for many years, compelling mechanistic evidence in support of this notion is still scant. This review sets into context the current knowledge about innate and adaptive immunological processes in psoriasis and other autoimmune or autoinflammatory diseases.

## Setting the Stage: Psoriasis as an Immune-Mediated Disorder

**Figure 1:**
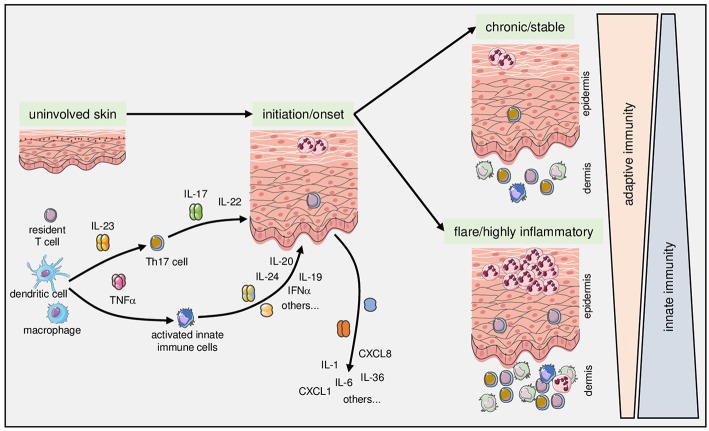
Complex fine-tuning of innate and adaptive immune mechanisms determines onset, course, and activity of psoriasis. As detailed in the text, intricate interactions between components of the innate (exemplified here by dendritic cells and macrophages) with components of the adaptive immune system (exemplified here by T cells) lie at the core of the pathophysiology of psoriasis. Once established, the relative contribution and fine-tuning of various mediators of adaptive and innate immunity determine the clinical manifestation toward chronic stable vs. highly inflammatory and/or pustular psoriasis.

If I was to name diseases that in recent years have increased our understanding of both adaptive and innate immune mechanisms on the one hand and have contributed decisively to the development of modern biological therapies on the other, then psoriasis would certainly occupy one of the top ranks. Psoriasis is currently viewed as a systemic chronic inflammatory disease with an immunogenetic basis that can be triggered extrinsically or intrinsically (1, 2). Research into its pathophysiology has led to impressive therapeutic improvements (3, 4). The disease is based on close interactions between components of the adaptive and the innate branches of the immune system (3, 5–9) (Figure 1). Since it was shown in the late 1970s that psoriasis can be ameliorated by cyclosporin A (10), it can no longer be seriously denied that T lymphocytes play a central role in the pathogenesis of this disease. This view is substantiated by numerous subsequent observations over the past four decades: psoriasis can be precipitated by bone marrow transplantation (11) and, similar to other autoinflammatory diseases, the disease is frequently associated with certain HLA expression patterns (7, 12–14). Drugs that specifically inhibit the function of T lymphocytes (such as CD2 blockade in the early days of biologics) can improve psoriasis (15). A therapeutic effect can also be achieved by interleukin (IL)-4, which pushes the cytokine milieu toward a T-helper (Th) cell 2-dominated immune response (16), probably through attenuation of Th17 function following diminished IL-23 production in antigen-presenting cells (17) and through induction of the transcription factor GATA3 (18, 19). IL-10 can also ameliorate psoriatic symptoms by modulating T cell functions (20). In addition, psoriasis-like skin inflammation in animal models can be initiated by certain CD4+ T cells (21–24), and T cells can induce psoriatic lesions in human skin xenografts (25, 26). Finally, the more recent discoveries that complexes of the antimicrobial peptide LL37 (a 37 amino acid C-terminal cleavage product of the antimicrobial peptide, cathelicidin) with own DNA or the melanocytic antigen ADAMTSL5 may function as autoantigens (27, 28), support the central role of T cells in the pathogenesis of psoriasis (29, 30).

## Autoimmune Processes in Psoriasis

### The Plot Thickens: Actual Auto-Antigens in Psoriasis

**Figure 2:**
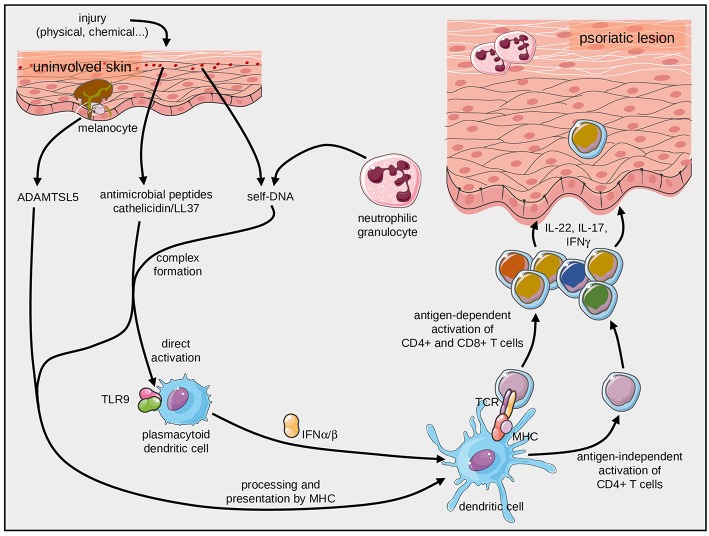
Initiation of psoriasis by antigen-dependent and antigen-independent immune mechanisms. Complexes of self-DNA with fragments of the antimicrobial peptide, cathelicidin, can stimulate plasmacytoid dendritic cells through TLR9. They can also be presented by HLA-C*06:02 molecules and specifically activate T cells through their TCR. Likewise, the melanocyte-derived ADAMTSL5 can activate pathogenic CD8+ T cells after presentation by HLA-C*06:02.

Pathogenic T cells in psoriatic skin lesions facilitate hyperproliferation of keratinocytes, influx of neutrophilic granulocytes, as well as production of other inflammatory cytokines, chemokines and antimicrobial peptides. They feature a Th17 signature, i.e., they express IL-17A, IL-22, and IFN-γ (3, 31, 32) (Figure 2). Dendritic cells maintain activation and differentiation of lesional Th17 cells primarily through secretion of IL-23 [reviewed in (8)].

In general, both HLA restriction and peptide specificity of a given T cell are determined by its T cell receptor (TCR) repertoire (33). Activation and clonal expansion of T cells occur upon antigenic stimulation. In the absence of foreign antigens, clonal T cell expansion is highly suggestive for autoimmunity in inflammatory diseases (34). Indeed, oligoclonal T cell expansion has been identified in psoriatic lesions in early well-designed studies (35–40) as well as in more recent investigations (41). It has been interpreted as an indicator for antigen-specific immune responses.

In psoriasis, oligoclonality of cutaneous T-cell populations is usually confined to lesional skin. This suggests that psoriasis is driven by locally presented antigens (35, 42–46). Likewise, the clonal TCRs arguably mark T cells which mediate the disease process. Several landmark publications during the past years lent support to this notion through identification of putative autoantigens in psoriasis.

Early concepts of autoimmune processes in psoriasis stemmed from the recognition of sequence homologies between keratinocyte structural proteins and streptococcal antigens (*S. pyogenes* in particular) (47–51). Mechanistic proof, however, is still lacking.

It was previously known that complexes of LL37 and self-DNA can activate dermal plasmacytoid dendritic cells (pDC) through toll-like receptor (TLR) signaling (52–54). These stimulate pDC then facilitate the psoriatic inflammatory cascade (52, 53, 55), a mechanism that is alluded to in more detail below. The activation *via* innate immune mechanisms was extended later by the finding that complexes of self-DNA and LL37 can also induce adaptive antigen-specific immune responses. Indeed, LL37 can trigger profound TCR and MHC (HLA-C^*^06:02)-dependent T-cell responses (28). It remains to be confirmed, however, that the LL37-related candidate peptides can be derived from the parent protein by antigen processing within the antigen presenting cell and then be presented by HLA-class I-molecules.

A more recent strategy to identify potential targets of pathogenic T-cells in psoriasis was based on the generation of T-cell hybridomas expressing the paired Vα3S1/Vβ13S1 TCR of clonal CD8^+^ psoriatic T cells of an HLA-C^*^06:02-expressing psoriasis patient (27). This elegant approach identified melanocytes as target cells of the psoriatic immune response (27). A peptide derived from ADAMTS-like protein 5 (ADAMTSL5) by proteasomal cleavage and post-cleavage trimming induced the specific immune response. The auto-antigenic function of melanocytic ADAMTSL5 was then confirmed by mutation and knock-down experiments. Moreover, peripheral lymphocytes of the majority of psoriasis patients but not individuals without psoriasis responded to ADAMTSL5 with production of IL-17 or IFNγ (27) (Figure 2). In contrast to LL37, which has been shown to activate both CD8^+^ cytotoxic T cells and CD4^+^ T helper cells, ADAMTSL5 appears to activate preferentially CD8^+^ T cells. Of note, both antigens are recognized by T cells when being presented by HLA-C^*^06:02, i.e., the most prominent psoriasis risk gene in the genome [located on PSORS1 (psoriasis susceptibility locus 1) on chromosome 6p21.3].

While the role of cellular adaptive immunity is becoming increasingly plausible, only recently autoantibodies, i.e., elements of humoral adaptive immunity, have been described in patients with psoriasis and psoriatic arthritis. Interestingly, these IgG are directed against (carbamylated/citrullinated) LL37 or ADAMTSL5 (56, 57). Since the serum concentrations of these antibodies were associated with the severity of psoriasis and since patients with psoriatic arthritis had higher serum levels, it is conceivable that a causal pathogenetic relationship and a contribution to systemic inflammation exist (56). It is also possible that the respective autoantibodies exert protective functions through scavenging autoantigens. However, their roles need to be clarified in future studies.

### The Other Side: Antigen Presentation by HLA Molecules in Psoriasis

While most, if not all, autoimmune diseases are linked with certain HLA alleles (58–60), HLA-C^*^06:02 is the predominant psoriasis risk gene (61–63). HLA class I molecules present short peptide antigens (8–10 amino acids) to αβ TCRs of CD8+ T cells. Such antigenic peptides are usually derived within the antigen presenting cell from (intracellular) parent proteins by proteasomal cleavage and loaded onto HLA-class I molecules. The HLA/peptide complex is then transported to the cell membrane where it can be recognized by CD8+ T cells (64, 65). Thus, HLA-class I-restricted immune responses are usually directed against target cells which produce the antigenic peptide.

HLA-C^*^06:02-presented non-apeptides (9 amino acids long) possess anchor amino acids at residues 2 (arginine) and 9 (leucine, valine, and less frequently methionine and isoleucine), along with a putative anchor at residue 7 (arginine). HLA-C^*^06:02 features very negatively charged pockets and thus binds to distinct positively charged peptides. Given that between 1,000 and 3,000 different self-peptides have been detected on HLA-C^*^06:02 under experimental conditions, multiple cellular proteins should be, in principle, presented by this HLA molecule and recognizable by CD8+ T cells (66, 67).

HLA-C^*^06:02, and other psoriasis-related HLA types such as HLA-C^*^07:01, HLA-C^*^07:02, and HLA-B^*^27 utilize identical anchor residues and present partially overlapping peptide residues (66, 67). Moreover, a negatively charged binding pocket is shared with another risk allele, HLA-C^*^12:03 (68, 69), resulting in similar functional domains and peptide-binding characteristics (67, 70). Thus, several HLA-class I types implicated in psoriasis appear to share similar peptide-binding properties. It is, therefore, conceivable that they can substitute for each other in conferring psoriasis risk. However, HLA-C^*^06:02 is the prototype allele within this spectrum and is associated with the highest risk for psoriasis.

### Supporting Acts: Indispensable Players in the Ensemble of Psoriasis Immunology

Autoantigen presentation alone does not suffice to induce the psoriatic cascade in genetically predisposed individuals. Rather, costimulatory effects of various gene products orchestrate the activation of the actual autoimmune response. Such risk gene variants modulate inflammatory signaling pathways (e.g., the IL-23 pathway), peptide epitope processing and/or Th/c17 differentiation (a selection of important factors is summarized in Table 1).

**Table 1:** Genetic factors implicated in psoriasis.

**Pathogenic function**	**Psoriasis-associated gene locus**
IL-23/IL-17A signaling	*IL23R*, interleukin-23 receptor
	*IL12B*, interleukin-12 subunit p40, also part of IL-23
	*IL12RB*, interleukin-12 receptor subunit beta 1, also termed IL-12Rβ1
	*IL23A*, interleukin-23 subunit alpha, p19
	*IL23R*, interleukin-23 receptor, a Janus kinase-2 associated type I cytokine receptor that activates STAT3 upon ligand binding
	*TYK2*, tyrosine-protein kinase 2, a Janus kinase family member, facilitates type I and II cytokine receptor and type I and III interferon signaling pathways, involved in innate and adaptive immune processes
	*STAT3*, signal transducer and activator of transcription 3, a central transcription factor in inflammatory processes
	*STAT5A/B*, signal transducer and activator of transcription 5A/B
	*SOCS1*, suppressor of cytokine signaling 1, a member of the STAT-induced STAT inhibitor family, negative regulator of cytokine signaling
	*ETS1*, a member of the E26 transformation-specific transcription factors, a negative regulator of Th17 cells
	*TRAF3IP2*, tumor necrosis factor receptor-associated factor 3-interacting protein-2, central role in response to inflammatory signals
	*KLF4*, Krüppel-like factor 4, influences NF-κB-mediated inflammatory pathways
	*IF3*, eukaryotic translation initiation factor 3
Effector T-cell function and differentiation	*ETS1*, E26 transformation-specific transcription factor 1, a negative regulator of Th17 cells
	*RUNX3*, runt-related transcription factor 3, a runt domain-containing transcription factor involved in regulation of many cellular processes
	*TNFRSF9*, tumor necrosis factor receptor superfamily member 9, CD137, costimulator of T cells
	*MBD2*, methyl-CpG-binding domain protein 2, binds to methylated DNA and regulates transcription from methylated gene promoters
	*IRF4*, interferon regulatory factor 4
Type I interferon and cytokine signaling	*ELMO1*, engulfment and cell motility protein 1
	*TYK2*, non-receptor tyrosine-protein kinase, a Janus kinase family member (see above)
	*SOCS1*, suppressor of cytokine signaling 1, a member of the STAT-induced STAT inhibitor family, negative regulator of cytokine signaling
	*IFIH1/MDA5*, Interferon-induced helicase C domain-containing protein 1/melanoma differentiation-associated protein 5, a CARD (caspase activation and recruitment domain) protein involved in IL-1 and IL-18 processing and in regulation of inflammation
	*RNF114*, ring finger protein 114, a ubiquitin ligase
	*IRF4*, interferon regulatory factor 4
	*RIG1/DDX58*, retinoic acid inducible gene I encoded by the DDX58 (DExD/H-Box Helicase 58) gene; contains a RNA helicase motif and a caspase recruitment domain (CARD), involved in regulation of immune responses
	*IFNLR1/IL28RA*, interferon lambda receptor 1, forms complex with IL10RB and interacts with IL-28A, IL-28B, and IL-29, involved in immune regulation
	*IFNGR2*, interferon gamma receptor 2, non-ligand-binding beta chain of the IFNγ chain
Regulation of NF-κB-associated inflammatory signaling pathways	*TNFAIP3*, TNFα induced protein 3, a ubiqitin-editing enzyme that inhibits NF-κB activation and TNF-mediated apoptosis; involved in the cytokine-mediated inflammatory responses
	*TNIP1*, TNFAIP3 interacting protein 1, a regulator of NF-κB activation
	*TYK2*, non-receptor tyrosine-protein kinase, a Janus kinase family member (see above)
	*REL*, reticuloendotheliosis oncogene, a NF-κB subunit (c-Rel) involved in apoptosis, inflammation and immune responses, SNPs are also associated with ulcerative colitis and rheumatoid arthritis
	*NFkBIA*, NF-κB inhibitor α, interacts with NF-κB/c-Rel involved in inflammatory responses.
	*CARD14*, caspase recruitment domain family member 14, a scaffold protein involved in cell adhesion, signal transduction and cell polarity, involved in NF-κB activation.
	*CARM1*, coactivator associated arginine methyltransferase 1, catalyzes methylation of histones and other chromatin-associated proteins, involved in regulation of gene expression
	*UBE2L3*, ubiquitin conjugating enzyme E2 L3, an E2 ubiquitin-conjugating enzyme, participates in ubiquitination of the p105 NF-κB precursor
	*FBXL19*, F-box and leucine rich repeat protein 19, an E3 ubiquitin ligase, binds to interleukin 1 receptor-like 1 and regulates its ubiquitination, associated with pulmonary inflammation and psoriasis
Antigen processing (N-terminal trimming)	*ERAP1*, endoplasmic reticulum aminopeptidase 1, involved in trimming of HLA class I-binding precursors enabling them to be presented on MHC class I molecules

*Most genetic associations have direct connections with immune functions implicated in the pathophysiology of psoriasis*.

These genetic variations create costimulatory signals which modulate innate and adaptive immune mechanisms and shape the proinflammatory environment. In sum and in conjunction with the appropriate HLA molecules and autoantigens, they may eventually exceed the thresholds for activation and maintenance of pathogenic autoimmune and autoinflammatory responses in psoriasis (29, 71). Likewise, regulatory mechanisms involving programmed death (PD)-1 signals have emerged recently as modulators of chronic inflammation in psoriasis (72). However, the complex interactions of various players are by no means fully understood. Therefore, they are listed here only as a whole.

The autoantigens described so far cannot fully explain the genesis of psoriasis. To give just one example of the latter notion: Psoriatic lesions can also occur in vitiligo foci that do not contain melanocytes (73, 74). Alterations of resident cell types such as vascular endothelial cells or the cutaneous nervous system are also involved in the disease process (75–77). Further research is certainly needed here.

## Shades of Gray: Crosstalk Between Adaptive and Innate Immunity in Psoriasis

In addition to the antigen-specific facilitation of inflammation in psoriasis, there are several strong connections to components of the innate immune system. The crosstalk between the innate and adaptive branches of the immune system in psoriasis is complex and can only be highlighted by a few selected examples. Its fine-tuning arguably determines the actual clinical correlate within the spectrum of the disease. Indeed, there is accumulating circumstantial evidence that in patients with stable and mild disease, mechanisms of adaptive immunity are more likely to be in the foreground, while innate mechanisms seem to be more important in patients with active severe disease, systemic involvement and comorbid conditions (78) (Figure 1). The impact on systemic comorbid diseases has been interpreted, at least in part, as a systemic “*spillover*” of innate inflammatory processes in severe psoriasis (78). Of course, such factors are not specific for psoriasis, but appear to account for a general inflammatory state in patients with severe psoriasis.

Patients with severe psoriasis have increased levels of inflammatory cytokines, CRP, fibrinogen, α2 macroglobulin or PAI-1 (plasminogen activator inhibitor-1) in the blood (79–81), they show transcriptomic, proteomic and metabolomic abnormalities (82) and there are connections with chronic stress (83) and biophysical properties of the skin (84).

The serum levels of inflammatory cytokines have been proposed as parameters for disease severity (85). Such general inflammatory markers are accompanied by increased numbers of Th1, Th17, and Th22 cells in patients with severe psoriasis (86, 87), which provides a direct link with autoimmune (adaptive) processes. Moreover, there is an increasing number of modulating factors, such as autoimmune reactivity to ribonucleoprotein A1 (HNRNPA1) (88), which impact on the course and severity of psoriasis.

One of the perhaps most vivid recent examples of how individual mediators influence the spectrum of psoriasis by shifting innate or adaptive immune processes comes from research on the interplay between IL-17- and IL-36-driven inflammation (89). The three IL-36 isoforms (IL-36α, β, and γ) belong to the IL-1 family and are upregulated in psoriatic skin (90, 91). They bind to the IL-36 receptor (IL-36R), thereby inducing transcription of several inflammatory mediators through NF-κβ activation. IL-36Ra (IL-1F9), an anti-inflammatory natural IL36R antagonist, is encoded by the *IL36RN* gene and is abundantly present in the skin of patients with psoriasis vulgaris, which may constitute part of the “*checks and balances*” that control the psoriatic inflammation (90, 92). Function-abrogating mutations in the *IL36RN* gene may result in unrestrained inflammatory effects of IL-36. Absence of IL-36Ra then leads to excessive neutrophil accumulation as observed in some cases of familial generalized pustular psoriasis (92–94). Palmoplantar pustular psoriasis, however, seems to be related to CARD14 variants rather than IL36RN mutations (95, 96).

While most cases of pustular psoriasis occur without such mutations (97), IL-36-related processes appear to contribute decisively to the actual clinical manifestation of specific psoriatic phenotypes: It has recently been shown elegantly that the skin of patients with psoriasis vulgaris differs significantly from that of patients with pustular psoriasis—in a sense, opposite ends of the spectrum of psoriasis: while in both forms numerous genes are expressed abnormally, these differentially expressed genes overlap only to a relatively small extent. In psoriasis vulgaris, genes involved in adaptive (T-cell-associated) immune processes predominate, whereas in pustular psoriasis processes of innate immunity (mainly neutrophil-associated) they are dysregulated. Interestingly, IL-36 seems to play an important role for the accumulation of neutrophilic granulocytes and a pustular phenotype of psoriasis (89). The balance between IL-36 and IL-17 seems to contribute—at least partially—to clinical symptoms of psoriasis vulgaris vs. psoriasis pustulosa. If this interpretation of the data is correct, then this would constitute a mechanism that regulates the fine-tuning between innate and adaptive immune processes.

## The IL-23/IL-17 Pathway Connects Innate and Adaptive Immunity in Psoriasis

The notion of psoriasis featuring elements of both antigen-specific autoimmunity and non-specific autoinflammation needs to be considered a bit more closely, in particular downstream of innate and/or adaptive activation processes. As of today, interfering with IL-17A or IL-23 are the most efficient treatment modalities against psoriasis (98). Indeed, the IL-23/IL-17 axis seems to be particularly well-suited to exemplify the intricate crosstalk between adaptive and innate immunity in psoriasis.

**Figure 3:**
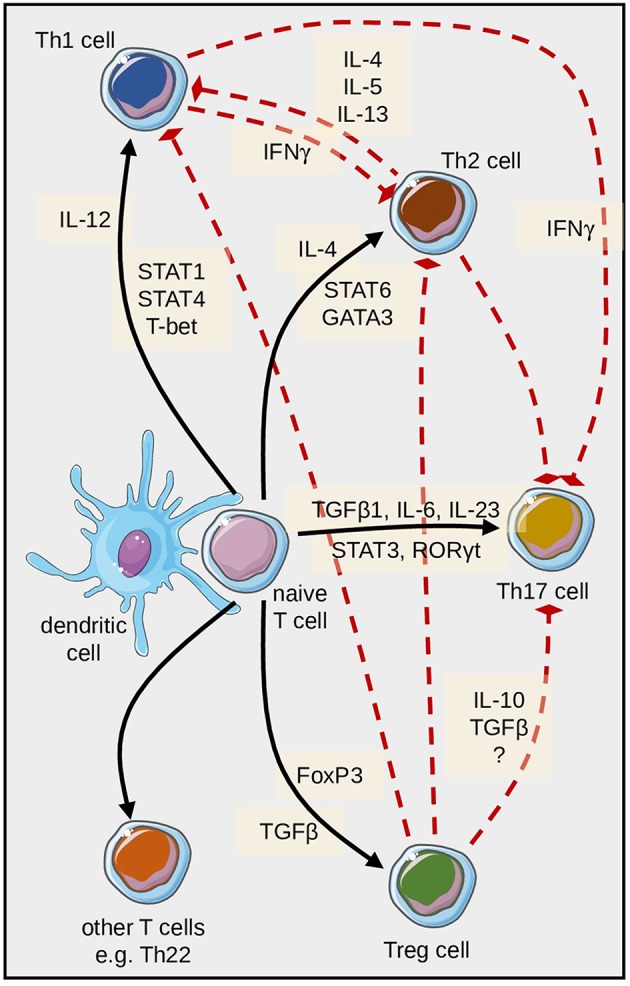
Differentiation of pathogenic T cells in psoriasis is embedded in a complex regulatory network. Naïve T cells can differentiate into several directions; this is mainly determined by the cytokines and transcription factors depicted here. In addition, various regulatory feedback mechanisms exist, some of which are schematically highlighted here with particular reference to Th17 cell differentiation and function.

Healthy human skin contains only a few IL-17-producing T cells (99), a population of CD4^+^ T cells distinct from the “classical” Th1 and Th2 cells. They were eponymously named for their production of IL-17 (100). In psoriasis (31, 101, 102), palmoplantar pustulosis (103) and other inflammatory disorders (104, 105), Th17 lymphocytes are vastly expanded and are thought to contribute decisively to the pathogenesis of these conditions (Figure 3). The resulting imbalance between Th17 and regulatory T cells (Treg) favors inflammation (106). The IL-17 production of T lymphocytes is further stimulated by activated keratinocytes, thus creating a positive feedback loop (107). Th17 cells are controlled by regulatory T cells through IL-10 (108). In psoriatic skin, IL-17A is considered the most relevant of the six known isoforms (102). IL-17A is not only secreted by CD4+ Th17 cells, but also by CD8+ T cells (109) and certain cells of the innate immune system including neutrophilic granulocytes (110–112), thus further highlighting the tight connection of innate and adaptive immunity in psoriasis. The presentation of IL-17 by neutrophil extracellular traps (NETs), which are generated upon activation of neutrophils in a clearly defined manner (113) and are prominently present in both pustular and plaque-type psoriasis (114), may also play a role (115).

In this context it should be mentioned that so-called tissue resident memory cells (T_rm_ cells) in psoriatic skin remain in the long term even after resolution of the lesions, which contribute as mediators of the local adaptive immune response to renewed exacerbations. Although the role of these cells is not yet fully understood, there is growing evidence of their pathogenic role in psoriasis and other chronic inflammatory diseases (116–118). T_rm_ cells in psoriatic lesions are CD8^+^ but lack CD49a (α1 integrin) expression; they predominantly generate IL-17 responses that promote local inflammation (119).

In addition to Th17 cells, T cells which produce both IL-17 and IFNγ (termed Th17/Th1-T cells) and IL-22-producing T cells can also be detected in psoriatic skin (31). Naive T cells express several cytokine receptors including the IL-23 receptor. DC-derived TGFß1, IL-1ß, IL-6, and IL-23 facilitate priming and proliferation of Th17 cells (120–122), while IL-12 assumes these functions for Th1 cells, and IL-6 and TNFα contribute to the programming of Th22 cells.

The balance of Th17 cells and Th1 cells appears to be critical for the pathogenesis of psoriasis (123) and other related conditions (124). There are several exogenous factors such as ultraviolet light or vitamin D3 (125, 126), or other cytokines like IL-9 (127) that can modulate Th17-dependent inflammation. Of note, IL-17A can also be produced independent of IL-23, e.g., by γδ T lymphocytes or invariant natural killer (iNKT) cells (128–130). However, it is not clear yet whether this alternative pathway impacts on the accrual and course of inflammatory disorders or potential undesired effects of either IL-23 or IL-17 inhibition.

In any case, the IL-23/IL-17 axis in psoriasis clearly illuminates the close interaction of the innate immune system (represented by IL-23-producing myeloid cells) with cells of the adaptive immune system (in this case Th17- and IL17-expressing CD8^+^ T-cells). Psoriasis could again serve as a “model disease” to clarify such relationships.

## Contribution of Resident Skin Cells to Immunological Processes in Psoriasis

Multiple genetic and environmental factors influence the immunopathology of psoriasis (131). The mechanisms leading to the first occurrence of psoriasis in predisposed individuals are only partly known. Infections with streptococci, medications such as lithium, antimalarials, or ß-blockers, or physical or chemical stress may trigger the disease. Minimal trauma can induce rapid immigration and activation of immune cells including T-cells and neutrophils (132), the so-called Köbner phenomenon (133, 134). Feedback loops between adaptive immune cells (T cells), innate immune cells (neutrophilic granulocytes, macrophages, dendritic cells), and resident skin cells (keratinocytes, endothelial cells) result in an amplification and chronification of the inflammatory response. Aspects of systemic inflammation in patients with severe psoriasis are thought to contribute to comorbid diseases (135).

Hyperproliferative keratinocytes in psoriatic plaques produce large amounts of antimicrobial peptides and proteins (AMP). These positively charged peptides, which have been termed alarmins, have strong proinflammatory properties. Most studies have addressed cathelicidin and its fragment, LL37, which is highly expressed psoriatic skin (136, 137). The positively charged LL37 can associate with negatively charged nucleic acids (DNA and RNA), thus forming immunostimulatory complexes. The free DNA required for such complexes probably comes from neutrophils (which form NETs) and damaged resident skin cells (e.g., traumatized keratinocytes). Plasmacytoid DC (pDC) and myeloid dendritic cells (DC) take up these complexes. Subsequently, RNA motifs stimulate toll-like receptors (TLR) 7 and 8, and DNA triggers TLR9 signaling (52, 138). Cytokines such as TNF, IL-23, and IL-12 are produced by TLR7/8-stimulated myeloid DC, while pDC make type I-interferons (IFNα), all of which fuel the psoriatic inflammation (131). A prominent role in psoriasis and other autoimmune diseases has been attributed to the so-called 6-sulfo LacNAc (slan) DC (139).

Several other skin-derived alarmins such as S100 proteins are inflammatory AMPs also implicated in the pathogenesis of psoriasis. Indeed, IL-17A induces the production of S100A7 (psoriasin) and S100A15 (koebnerisin) by keratinocytes (140, 141). Likewise, myeloid cells and keratinocytes produce the calgranulins, S100A8 and S100A9 [also termed myeloid-related protein (Mrp) 8 and Mrp14], both of which induce T-cell mediated autoimmune reactions and inflammatory changes in keratinocytes (142, 143). Similar to LL37, human ß-defensin (HBD) 2 and HBD4 bind DNA, trigger TLR9 and stimulate pDC (144). Innate immune sensing is also facilitated by IL-26 bound to self-DNA (145).

Activated DC in turn can program the differentiation of naive T into pathogenic T cells [reviewed in (8)]. Neutrophilic granulocytes, too, release AMP, inflammatory cytokines, proteases, free oxygen radicals, and NETs, all of which have been implicated in the inflammatory cascade in psoriasis (8, 114).

## Not Alone: Relations and Similarities of Psoriasis With Other Autoimmune and Autoinflammatory Disorders

The highlights outlined so far show that both adaptive and innate immune processes contribute to psoriasis. Their balance and fine-tuning seem to determine the development of certain clinical forms of the disease, but also organ-specific manifestations. On the one hand, the outlined long-term systemic inflammatory processes probably contribute to the pathogenesis of important metabolic, cardiovascular, and mental concomitant diseases. In these areas, the evidence of a causal relationship is becoming increasingly clear and numerous publications prove this. A more detailed overview can be found elsewhere in this thematic focus. On the other hand, the contoured adaptive and innate immune mechanisms are not specific for psoriasis. Rather, many of them have been found—in varying degrees and weightings—in a whole range of other autoimmune and autoinflammatory diseases. In any case, although this interplay of different components of the immune system is certainly not yet fully understood, parallels with other chronic inflammatory and autoimmune diseases emerge that underpin our current view of psoriasis as a systemic disease.

Indeed, the prevalence of several autoimmune and/or autoinflammatory diseases including rheumatoid arthritis, celiac disease, Crohn's disease, multiple sclerosis, systemic lupus erythematosus, vitiligo, Sjögren's syndrome, alopecia areata, or autoimmune thyroiditis appears to be increased in patients with psoriasis compared to that in healthy controls (146, 147). Several other and more uncommon associations have also been reported (148). Such associations have been attributed to certain genetic and immunological similarities and “overlaps” (146, 149). Three such disease complexes associated with psoriasis, i.e., rheumatoid arthritis, Crohn's disease and systemic lupus erythematosus, will be briefly discussed as examples.

Psoriasis susceptibility 1 candidate gene 1 *(PSORS1C1)*, a gene thought to be involved in IL-17 and IL-1β regulation, is increased in immune cells from patients with rheumatoid arthritis (150). Moreover, aberrant expression of runt-related transcription factor 1 (*RUNX1*) has been implicated in defective regulation of sodium-hydrogen antiporter 3 regulator 1 (*SLC9A3R1*) and N-acetyltransferase 9 (*NAT9*) in both psoriasis and rheumatoid arthritis (151–153). Polymorphisms of the IL-23R gene have also been implicated in both diseases, which further underscores the general relevance of the IL-23/IL-17 axis (154). TNFα-induced protein 3 (*TNFAIP3*), which negatively regulates NF-κB signaling, is another gene thought to be involved in rheumatoid arthritis and psoriasis alike, but also in Crohn's disease, celiac disease, and systemic lupus erythematosus (155, 156).

Similar functional imbalances between Th17 and regulatory T cells (Tregs) as well as similar central cytokines including TNFα, IL-23, and IL-17A, but also IL-1β, IL-6, IL-17F, and IL-21 contribute to both diseases (131, 157–159). Such striking parallels result in the response of both disorders to the same therapies.

Similar to rheumatoid arthritis, Crohn's disease is significantly more prevalent in patients with psoriasis compared to healthy controls and *vice versa* (160–164). Moreover, considerable genetic overlap exists between both diseases as exemplified by seven mutual susceptibility loci (165). Genes involved in the same way include some relevant for the IL-23/I-17 axis such as *IL23R, IL12B*, and *TYK2* (166–169).

**Figure 4:**
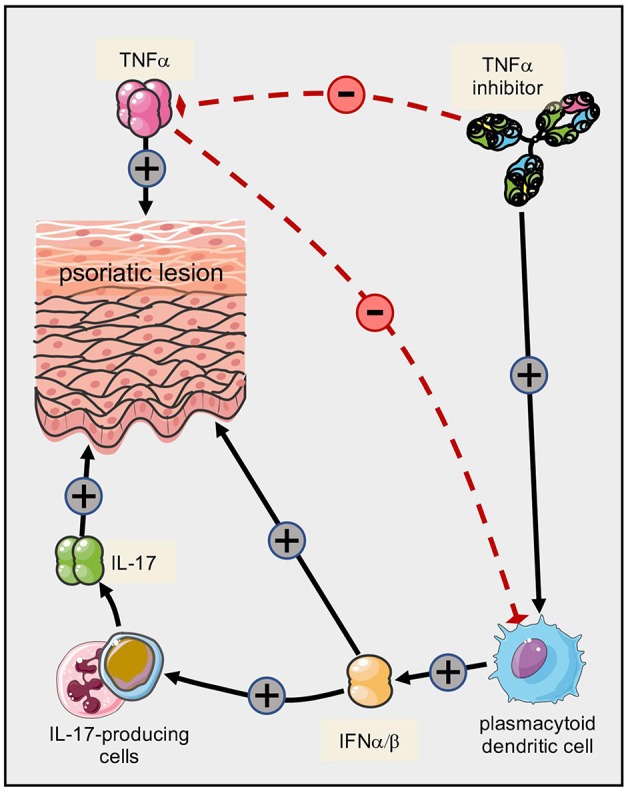
Paradoxical psoriasis triggered by TNF inhibitors in predisposed individuals. Several cytokines contribute to the pathogenesis of psoriatic skin lesions, with TNFα and IL-17A playing prominent roles. However, TNFα also exerts an inhibitory effect on plasmacytoid dendritic cells. Upon therapeutic inhibition of TNFα, this inhibitory effect is abrogated and the resulting shift toward increased production of type I interferons fuels the secretion of IL-17. It is conceivable that additional mechanisms contribute to the shift of cytokines ultimately resulting in “paradoxical” psoriatic lesions.

One of the first immunological parallels found between psoriasis and Crohn's disease was the central pathogenic role of TNFα (170–172). Hence, TNFα inhibitors ameliorate both disorders (172–175). Paradoxical induction of psoriasis in patients treated with TNFα inhibitors has been attributed to shifts within the balance of TNFα and type I interferons (IFNα) with impact on plasmacytoid dendritic cells (176–181) (Figure 4). The pathophysiology of such paradoxical reactions in other immunomodulating settings is less clear (182, 183). The composition of the inflammatory infiltrate (T cells, macrophages, dendritic cells and neutrophilic granulocytes) as well as inflammatory mediators (IFNγ, IL-12, IL-6, IL-17) are conspicuously similar in psoriasis and Crohn's disease (131, 184). A dysregulated balance between Th17 cells and CD4+CD25high Foxp3+ Tregs is thought to lie at the core of both diseases (101, 157, 185–187). In addition, there may even be IL-17 producing Tregs in lesions of both Crohn's disease and psoriasis (188, 189), suggesting differentiation of Tregs toward a pro-inflammatory phenotype. However, a putative protective role of IL-17 in Crohn's disease (190) may explain, at least in part, the worsening of gut inflammation in some cases upon inhibition of IL-17A (191).

Increased expression of IL-6 has been demonstrated in psoriatic plaques and inflamed intestinal mucosa alike (192, 193). IL-6 signaling induces STAT3 phosphorylation, which leads to relative resistance of effector T cells toward Tregs (194, 195).

The association of psoriasis and systemic lupus erythematosus is uncommon and controversially discussed (196, 197). However, dysfunctional interaction of RUNX1 with its binding site due to nucleotide polymorphisms links psoriasis not only with rheumatoid arthritis but also with systemic lupus erythematosus (153, 198, 199). RUNX1 binding on chromosome 2 is defective in some patients with SLE, while RUNX1 binding on chromosome 17 seems to be altered in some psoriasis patients.

TNF receptor-associated factor 3 Interacting Protein 2 (*TRAF3IP2*) has been described as a genetic susceptibility locus for psoriasis and appears to facilitate IL-17 signaling in both psoriasis and systemic lupus erythematosus (200–205). On the cellular level, psoriasis and systemic lupus erythematosus share impaired Treg functions (157, 192, 206, 207), thus suggesting that similar genetic and immune alterations govern pathological immune reactions in both psoriasis and systemic lupus erythematosus.

In summary, psoriasis shows elements of both autoimmune and autoinflammatory mechanisms, whose fine-tuning determines the actual clinical symptoms within the broad spectrum of the disease. Given that psoriasis is a systemic disease that shares conspicuous genetic and immunological similarities with other autoimmune and autoinflammatory disorders, it may serve as a model disorder for research into general mechanisms of such complex immunological regulations.

## Author Contributions

The author confirms being the sole contributor of this work and has approved it for publication.

### Conflict of Interest Statement

The author declares that the research was conducted in the absence of any commercial or financial relationships that could be construed as a potential conflict of interest.
